# Effects of exogenous lactic acid bacteria and maize meal on fermentation quality and microbial community of *Orychophragmus violaceus* silage

**DOI:** 10.3389/fmicb.2023.1276493

**Published:** 2023-09-21

**Authors:** Mingli Zheng, Peichun Mao, Xiaoxia Tian, Lin Meng

**Affiliations:** Institute of Grassland, Flowers and Ecology, Beijing Academy of Agriculture and Forestry Sciences, Beijing, China

**Keywords:** *Orychophragmus violaceus*, silage, lactic acid bacteria, fermentation quality, bacterial community

## Abstract

*Orychophragmus violaceus* is a local Brassicaceae in China, while most of it is directly mowed and discarded after the ornamental period. In order to develop forage resources, this study firstly evaluated the potential preservation of *O. violaceus* silage. *O. violaceus* was harvested at full-bloom stage, and ensiled without (CK) or with maize meal (Y5), lactic acid bacteria inoculant (Z) and compound additive (Y5Z) for 60 d. Results of chemical and microbiological analysis showed that a large amount of lactic acid was produced and the final pH value was below 4.1 in silages regardless of additive application. CK silage was well preserved as indicated by the low levels of dry matter loss and butyric acid content, and the predominant genus were identified as *Enterococcus* and *Pediococcus*. Y5 silage had potential health risks for humans and animals as seen by frequent occurrence of pathogenic bacteria *Clostridium* and *Achromobacter*. Z and Y5Z silages were poorly preserved, resulting in great dry matter loss and butyric acid content. Considering the abundant acetic acid production, the dominant *Lactobacillus* might possess a heterofermentative pathway in Z and Y5Z silages. In conclusion, *O. violaceus* has the potential to be long stored as silage because of its sufficient water-soluble carbohydrates, while exogenous lactic acid bacteria and maize meal generally provided little positive effect. In future research, efficient homofermentative *Lactobacillus* strains were suggested to be screened to further enhance the ensiling process of *O. violaceus* silage.

## Graphical abstract

**GRAPHICAL ABSTRACT:**
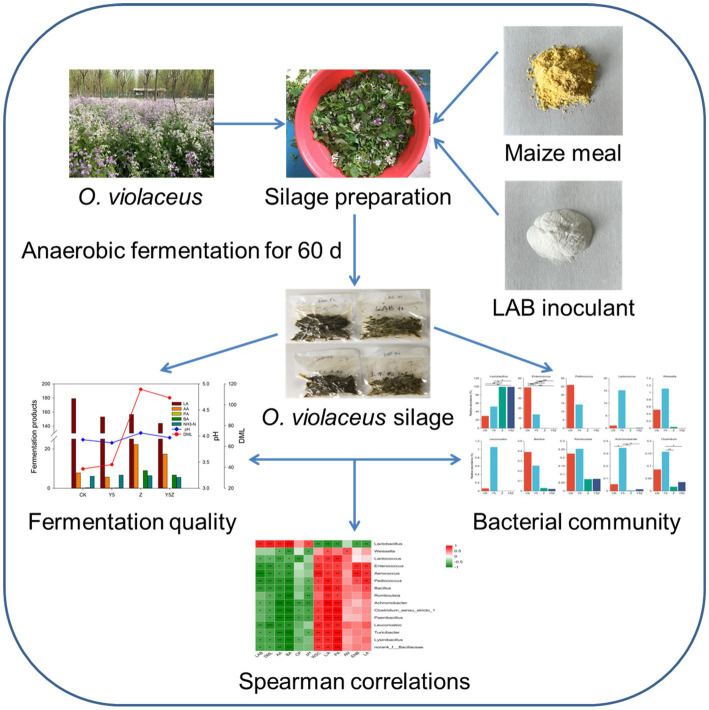

## 1. Introduction

*Orychophragmus violaceus* (Brassicaceae) is a popular ornamental plant with a local flavor and natural charm which is endemic to China (Zhou et al., 2008), with strong environmental adaptability and breeding ability, and is widely distributed in various regions of China (Hang, 2022). Starting from the February of the lunar calendar, the blooming *O. violaceus* spread from the south to the north of China, creating a spectacular and eye-catching scene. However, the greatest ornamental period of full flowered *O. violaceus* was only 20 to 30 d. After the ornamental period, a small part of *O. violaceus* is used as green manure to provide nutrients for the soil (Cao et al., 2014), while most of it is directly mowed and discarded, resulting in a large amount of waste of biomass resources.

*O. violaceus* is a common wild vegetable that has large tender stems and highly nutritious leaves. In addition, this plant has a long history as a traditional Chinese medicine for anti-tumor, anti-inflammatory, anti-bacterial and liver-protecting properties, as it is rich in bioactive components (Xu et al., 2022). Therefore, we speculated that the application of *O. violaceus* as feed may be favorable for preserving and improving animal performance. Especially, in the context of the current global shortage of feed resources, expansion of the forage sources will make an important contribution to enhance the level of national food security and to maintain a sustainable and economically viable livestock system (Ni et al., 2017). However, *O. violaceus* harvest is seasonal with high accumulation, if it is not consumed in a short period of time by animals, it gets spoilage due to the high moisture content.

Ensiling is a practicable technique for long-term preservation of moist crops, for the preparation of well-preserved silage, a rapid development of the lactic acid (LA) fermentation is necessary to reduce the pH and inhibit the growth of inefficient and spoilage microorganisms (Zheng et al., 2018). In addition to various environmental factors, such as method of ensiling, climate, stage of maturation and wilting, lactic acid bacteria (LAB) and water-soluble carbohydrates (WSC) are crucial factors controlling the ensiling process. LAB inoculants have become the most commonly used silage additives even for direct-cut crops, because the LAB that are the essential microflora for spontaneous silage fermentation are orders of magnitude lower in population than other groups of microorganisms on the crop at ensiling (Muck, 2010). Homofermentative *Lactobacillus plantarum* was often used to improve the ensiling process as it allows for a rapid pH decrease, while usage of heterofermentative *L. buchneri* could also be beneficial as it produces sufficient acetic acid (AA) to depress aerobic deterioration by inhibiting growth of yeast and fungi (Zhang et al., 2009; Guo et al., 2018). However, to our knowledge, limited information is available on the role of LAB inoculant in *O. violaceus* silage. Maize meal with wide source and low cost is also commonly used to regulate the raw material properties of wet forage for high quality silage preparation because of its high dry matter (DM) and WSC contents (Niu et al., 2018).

Silage fermentation is a complex biochemical process with the participation of many microorganisms. Considering the fact that microorganisms are the key factors driving ensiling process, monitoring of the ensiling process with respect to changes in the chemical and microbial compositions would be helpful for thoroughly understanding and improving the ensiling process (Zheng et al., 2017; Huang et al., 2023). Therefore, this study first aimed to evaluate the fermentation quality and bacterial community of *O. violaceus* silages with or without exogenous LAB inoculant and maize meal. The results will provide a scientific theoretical basis for *O. violaceus* silage preparation.

## 2. Materials and methods

### 2.1. Silage preparation

*Orychophragmus violaceus* was cultivated under an ash (*Fraxinus chinensis*) forest (40°9′N, 116°57′E) located at Beijing, China, and harvested artificially at full-bloom stage after the ornamental period. The harvested *O. violaceus* was chopped to length of 2–3 cm using a forage cutter, mixed manually, and divided randomly into four treatments: (i) addition with an equal volume of distilled water (Control, CK); (ii) addition with maize meal at an application rate 50 g/kg fresh matter (FM)(Y5); (iii) addition with LAB inoculant at a concentration of 10^6^ cfu/g FM (Z); and (iv) addition with maize meal at an application rate 50 g/kg FM and LAB inoculant at a concentration of 10^6^ cfu/g FM (Y5Z). Maize meal used in this study was from a local supermarket, its chemical and microbial compositions were shown in Table 1. The applied LAB inoculant was ZHUANGLEMEI (Sichuan Gao Fu Ji Biological Technology Co., Ltd., Chengdu, China), consisting of *L. plantarum* and *L. buchneri* with a ratio of 7:3. *O. violaceus* material was mixed homogenously with additives, packed manually into plastic film bag silos (Hiryu KN type, 180 × 260 mm; Asahikasei, Tokyo, Japan) and vacuumed tightly. Silos were prepared in triplicate and stored at ambient temperature (20–35°C) for 60 d of ensiling.

**Table 1:** Chemical compositions and microbial counts of fresh *Orychophragmus violaceus* and maize meal.

Item	*O. violaceus*	Maize meal	SEM	*p*-value
Chemical compositions (g/kg DM, unless stated otherwise)				
DM (g/kg FM)	143.95b	906.72a	170.564	<0.001
CP	215.42a	103.20b	25.132	<0.001
WSC	124.93a	10.25b	25.664	<0.001
NDF	294.42a	24.50b	60.503	<0.001
ADF	228.58a	5.95b	49.852	<0.001
Microbial counts (log_10_cfu/g FM)				
LAB	4.10	3.93	0.094	0.414
Aerobic bacteria	5.15a	3.83b	0.296	<0.001
Enterobacteria	6.13a	4.11b	0.455	<0.001
Yeast	4.11a	2.40b	0.382	<0.001

DM, dry matter; FM, fresh matter; CP, crude protein; WSC, water-soluble carbohydrates; NDF, neutral detergent fiber; ADF, acid detergent fiber; LAB, lactic acid bacteria; cfu, colony-forming units; 2.40, i.e., log_10_ 250 cfu/g, microbial count below the detection limit (500 cfu/g) is assigned a value corresponding to half of the detection limit, numerically for analysis of variance. SEM, standard error of mean. Values with different letters in the same rows are significantly different at level of *p* < 0.05.

### 2.2. Chemical composition analysis

Fermentation products of silage were determined from cold-water extracts. Wet silage (10 g) was homogenized with 90 mL of sterilized distilled water, and then filtered through four layers of medical gauze and a qualitative filter paper. The pH was measured with a glass electrode pH meter (S20K; Mettler Toledo, Greifensee, Switzerland), and ammonia nitrogen (NH_3_−N) content was determined by the method of Broderick and Kang (1980). The filtrate was further processed with a dialyser of 0.22 μm to determine organic acids contents as described by Zhang et al. (2020). Briefly, LA was analyzed by ion chromatography (Dionex ICS-2500, Dionex instruments, California, CA) equipped with an InoPac AS11-HC analysis column (4 × 250 mm), an InoPac AS11-HC protect column (4 × 50 mm), and an ASRS ULTRA II 4 mm suppressor. The sampling amount was 25 μL, the column temperature was 30°C, the mobile phase was 50 mmoL/L sodium hydroxide solution at a flow rate of 0.80 mL/min. AA, propionic acid (PA) and butyric acid (BA) contents were determined with GC 3420 gas chromatograph (Agilent Tech Inc., Dionex, FTC, Palo Alto) fitted with HP-INNO wax capillary column (30 m × 0.32 mm).

Silage samples were dried at 65°C for 48 h to determine the DM content, and then ground through 0.20 mm-mesh sieves for analysis of chemical components. The crude protein (CP) was determined by the method of AOAC (1990). Both neutral detergent fiber (NDF) and acid detergent fiber (ADF) were determined using an ANKOM 2000 fiber analyzer (Ankom Technology, Fairport, NY) by the method of Vansoest et al. (1991). The WSC was determined by the method of Owens et al. (1999).

### 2.3. Microbial population analysis

For microbial population analysis, wet silage samples (10 g) were blended with 90 mL of sterilized water and serially diluted (10^-1^ to 10^-5^) in sterilized water. The numbers of LAB were measured by plate count on de Man, Rogosa and Sharpe Agar (Difco Laboratories, Detroit, MI) incubated at 37°C for 48 h under anaerobic condition (Anaerobic box, TE-HER Hard Anaerobox, ANX-1; Hirosawa Ltd., Tokyo, Japan). The microbial counts for aerobic bacteria were determined on Nutrient Agar (Nissui Ltd., Tokyo, Japan) incubated at 37°C for 48 h. The numbers of Enterobacteria were determined on Blue Light Broth Agar (Nissui Ltd) incubated at 37°C for 48 h. Yeasts were counted on Potato Dextrose Agar (Nissui Ltd.) incubated at 30°C for 48 h. The colonies were counted from the plates at appropriate dilutions, and the number of colony forming units is expressed per gram of FM (Wang et al., 2016).

### 2.4. Bacterial community analysis

The extraction of total genomic DNA from each silage sample was performed using the FastDNA™ SPIN Kit for Soil and the FastPrep Instrument (MP Biomedicals, Santa Ana, CA) as we described previously (Zheng et al., 2017). The V3–V4 regions of the bacterial 16S rRNA genes were amplified using the primers pairs 338F (5′-ACTCCTACGGGAGGCAGCAG-3′) and 806 R (5′-GGACTACHVGGGTWTCTAAT-3′). TruSeq™ DNA Sample Prep Kit (Illumina, San Diego, CA) was used to construct the sequencing library following manufacturer’s recommendations. DNA libraries were then paired-end sequenced on the Illumina MiSeq PE300 platform (Illumina). Both of PCR amplification of 16S rRNA genes and MiSeq sequencing were carried out by the Majorbio Biopharm Technology Co., Ltd. (Shanghai, China). The sequencing data were submitted to the NCBI Sequence Read Archive database (accession: PRJNA1002122).

The raw data from sequencing were merged using FLASH (version 1.2.11, https://ccb.jhu.edu/software/FLASH/index.shtml), followed by removing low-quality sequences and chimeric sequences using FASTP (version 0.19.6, https://github.com/OpenGene/fastp), respectively. A default similarity level of 97% was used to cluster sequences into individual operational taxonomic units (OTUs) using UPARSE (version 7.0.1090, http://drive5.com/uparse/). The representative sequence from each clustered OTU was used to taxonomic classification in the SILVA database (Release 138, https://www.arb-silva.de/) at a minimum confidence cut-off of 0.7 using the RDP Classifier (version 2.13, https://sourceforge.net/projects/rdp-classifier/). Bacterial alpha diversity indices, including Sobs, Ace, Chao1, Shannon, Simpson and Coverage, were estimated using MOTHUR (version 1.30.2, https://www.mothur.org/wiki/Download_mothur). To explore relationships between the microbial community and fermentation products, Spearman’s rank correlation matrix was generated by calculating the Spearman’s correlation coefficient. The level of high significance was set to *p*-value of less than 0.05 with |r| > 0.40. The correlation matrix was visualized as a heat map produced at the genus level using the R program (version 3.6.0, https://www.r-project.org) (Li et al., 2020). Bioinformatics analysis was performed on the online platform of Majorbio Cloud Platform.^1^

### 2.5. Statistical analysis

All microbial counts were log_10_ transformed to obtain log-normal distributed data. To calculate averages, the values below the detection level (500 cfu/g) were assigned a value corresponding to half of the detection level (i.e., 250 cfu/g). Data for chemical compositions, microbial counts and bacterial community indices were analyzed by a one-way ANOVA using the GLM procedures of SAS 9.1 (SAS Institute, Inc., Cary, NC), and the Tukey’s test was used for multiple comparisons at 5% significant level.

## 3. Results and discussion

### 3.1. Characteristics of fresh *Orychophragmus violaceus*

The microbial and chemical compositions of *O. violaceus* prior to silage preparation are shown in Table 1. The CP content was as high as 215.42 g/kg DM, which was comparable to that of alfalfa (Zheng et al., 2017), indicating that *O. violaceus* is a promising source of protein for animal feed. Ensiling fermentation by microbial decomposition of WSC can produce organic acids, thereby lowering the pH value and inhibiting the proliferation of deteriorating bacteria (Huang et al., 2023). In this study, the WSC content of 124.93 g/kg DM of *O. violaceus* was sufficient (>50 g/kg DM) for LA fermentation. However, the epiphytic LAB of forages do not always reduce pH rapidly because the initial load can be too low or fast acidifying homofermentative species may be absent (Zheng et al., 2018). Therefore, LAB inoculants were used to enhance the ensiling process of *O. violaceus* based on its insufficient (<10^5^ cfu/g FM) epiphytic LAB counts of 10^4^ cfu/g FM. A lower DM content of 143.95 g/kg FM (<300 g/kg FM) is an additional inhibitory factor to LA fermentation, as high moisture content might dilute WSC concentration resulting in BA fermentation (Pahlow et al., 2003).

### 3.2. Chemical compositions of *Orychophragmus violaceus* silage

For CK silage (Table 2), the DM content (139.26 g/kg FM) was comparable with fresh material and the CP content was similar to that of alfalfa silage (Zheng et al., 2018; Guo et al., 2020), therefore, this study further confirmed the potentiality for *O. violaceus* silage as protein resource. As expected, the residue WSC content in Z and Y5Z silages was lower (*p* < 0.05) than that in CK and Y5 silages, so LAB inoculation promoted WSC consumption for acids production (Niu et al., 2018). The lowest (*p* < 0.05) NDF and ADF contents occurred for Y5 and Y5Z silages in relative to CK. We attributed the lower NDF and ADF contents in maize meal-added silages to a result from acidic hydrolysis and/or fibrinolytic enzyme production by microorganisms during silage fermentation. However, although we could not classify the behind reason clearly, the NDF and ADF contents increased (*p* < 0.05) when LAB inoculant was added alone in Z silage compared to CK.

**Table 2:** Chemical compositions of *Orychophragmus violaceus* silages with or without LAB and maize meal.

Item	CK	Y5	Z	Y5Z	SEM	*p*-value
DM (g/kg FM)	139.26c	178.72a	128.84d	167.06b	6.145	<0.001
CP (g/kg DM)	223.43b	188.57c	238.90a	221.07b	4.330	<0.001
WSC (g/kg DM)	26.17a	22.37a	8.47c	12.68b	1.612	<0.001
NDF (g/kg DM)	307.07b	230.23c	333.03a	221.08c	10.380	<0.001
ADF (g/kg DM)	247.98b	186.88c	271.08a	178.37c	8.498	<0.001

CK, control; Y5, with maize meal; Z, with LAB; Y5Z, with LAB and maize meal. Values with different letters in the same rows are significantly different at level of *p* < 0.05.

### 3.3. Fermentation characteristics, fermentation losses and microbial counts of *Orychophragmus violaceus* silage

The final pH values in all silage samples were below 4.1 (Table 3), suggesting a potential rapid acidification process of *O. violaceus* silage. LA production in CK silage was 179.13 g/kg DM, which was higher than that in alfalfa silage of 12.2–82.4 g/kg DM (Zheng et al., 2017) and whole crop maize silage of 80–120 g/kg DM (Wang et al., 2022). The effects of maize meal and LAB inoculant on *O. violaceus* silage fermentation were unexpected, regarding the lower (*p* < 0.05) LA concentration in Y5, Z and Y5Z silages compared to CK. The limited effect of maize meal and LAB inoculant on LA production might partly be related to the sufficient fermentable carbohydrates as well as other bioactive compounds favoring the growth of native LAB in *O. violaceus*. Forage crops used for silage preparation vary in DM content, carbohydrates, buffering capacity and carry a microflora of unknown magnitude and composition, these factors plus the establishing and maintaining a strictly anaerobic environment often determine the conversion of plant sugars to LA (Pahlow et al., 2003).

**Table 3:** Fermentation characteristics, fermentation losses and microbial counts of *Orychophragmus violaceus* silages with or without LAB and maize meal.

Item	CK	Y5	Z	Y5Z	SEM	*p*-value
Fermentation characteristics						
pH	3.93	3.87	4.06	3.97	0.033	0.236
LA (g/kg DM)	179.13a	153.16b	156.61b	143.95b	4.364	0.004
AA (g/kg DM)	7.79b	5.67b	22.22a	17.34a	2.215	0.001
L/A	23.19a	27.22a	7.59b	8.40b	2.703	<0.001
PA (g/kg DM)	0.09a	0.11a	0.00c	0.04b	0.013	0.001
BA (g/kg DM)	0.25b	0.15b	8.96a	6.73a	1.319	0.004
Fermentation losses						
DML (g/kg DM)	38.49b	42.66b	114.83a	106.65a	11.162	<0.001
NH_3_−N (g/kg TN)	6.09b	6.64a	6.42ab	5.58c	0.104	<0.001
Microbial counts (log_10_ cfu/g FM)						
LAB	5.56b	6.11b	7.21a	7.37a	0.061	<0.001
Enterobacteria	2.40	2.40	2.40	2.40	0	–
Yeasts	2.91b	4.78a	2.40b	2.40b	0.315	<0.001
Aerobic bacteria	3.90	3.99	3.20	3.04	0.186	0.165

LA, lactic acid; AA, acetic acid; L/A, lactic to acetic acid ratio; PA, propionic acid; BA, butyric acid; DML, dry matter loss; NH_3_−N, ammonia nitrogen; TN, total nitrogen. Values with different letters in the same rows are significantly different at level of *p* < 0.05.

AA (pKa = 4.76) is weaker for silage acidification compared to LA (pKa = 3.86) (Contreras-Govea et al., 2013; Kung et al., 2018). Similar to previous results found in corn silage (Wang et al., 2022), a combination inoculant of *L. buchneri* and *L. plantarum* increased (*p* < 0.05) the AA concentration in Z and Y5Z silages compared to that in CK and Y5 silages. The most common reason for an inoculant not producing an effect is competition from the epiphytic LAB population, therefore, we hypothesized that *L. plantarum* strain applied in this study could not adapt to the nutritional characteristics of *O. violaceus*, or they are more sensitive than *L. buchneri* to the bioactive ingredients of *O. violaceus*. This shows the importance of the origins of the LAB inoculants when selecting them, and many studies show that the best isolates for a specific crop would come from that crop itself (Wang et al., 2006). Undetectable or traceable PA concentration was observed in all silages, while high BA (>5 g/kg DM) production was detected in Z and Y5Z silages. BA was undesirable in well-fermented silage, as the presence of this acid usually indicates the clostridial fermentation (Zheng et al., 2020). Although we do not have direct evidence, the high BA production in Z and Y5Z silages might be due to the slow acidification process at the initial fermentation stage, resulting from the competition for nutrients between the inoculated *L. buchneri* and native LAB.

NH_3_−N is undesirable during ensiling fermentation as it is an important indicator of protein degradation. In this study, NH_3_−N concentration in all *O. violaceus* silages ranged from 5.58 to 6.64 g/kg total nitrogen (TN), which was less than normal level of NH_3_−N (100–150 g/kg TN) in high moisture legume silage (Kung et al., 2018). It is considered that NH_3_−N accumulation during ensiling was attributed to the growth of undesirable bacteria (such as Enterobacteria, Bacillus and Clostridia) and/or the activities of inherent plant proteolytic enzymes (Li et al., 2022). Therefore, the decreased NH_3_−N content which indicated an improvement in protein quality in *O. violaceus* silage might be due to the fact that the lowered pH limited the proteolytic activity of microorganisms and plant proteolytic enzymes (Bao et al., 2023). Whatever strategies are developed, they must be consistent with well accepted practices that minimize silage storage losses (Kung et al., 2018). The dry matter loss (DML) associated with silage fermentation are primarily from carbon dioxide and volatile fatty acids production, and these losses typically are in the range of 20 to 40 g/kg DM (Borreani et al., 2018). In this study, the DML in CK and Y5 silages was similar to the normal level, while the values in Z and Y5Z silages were higher than 106.65 g/kg DM. The amount of DML from fermentation depends on the dominant microbial species and the substrates fermented, and it was reported that DML of 48–328, 170, 511 and 489 g/kg DM could be occurred because of fermentation caused by heterofermentative LAB, Enterobacteria, Clostridia and yeasts, respectively (Borreani et al., 2018).

Due to the high concentration and strong adaptability of LAB inoculants, it was hypothesized that inoculant strains are highly competitive over the epiphytic LAB as well as other epiphytic bacteria on crops, dominating silage fermentation (Muck, 2013). In fact, the LAB counts in Z and Y5Z silages were higher (*p* < 0.05) than that in CK and Y5 silages. Proper management during silage preparation is an important and efficient way for controlling the development of undesirable microorganisms (Franco et al., 2022). Enterobacteria decreased to below the detectable level in all silages. The yeast numbers also dropped below the threshold of detection in Z and Y5Z silages, might being due to the antifungal inhibition by AA. The aerobic bacteria decreased by an order of magnitude after ensiling fermentation, although the final numbers were still higher than 10^3^ cfu/g FM in all silages. The suppression of detrimental microorganisms in *O. violaceus* silages with or without additives might partly be related to efficient acidic environment as well as antimicrobial bioactive components found in *O. violaceus*.

### 3.4. Bacterial community of *Orychophragmus violaceus* silage

The alpha diversity estimates of bacterial community are presented in Table 4. Coverage index in all silages was above 0.99, indicating that the depth of high-throughput sequencing could represent the profile of bacterial community. As expected, Sobs index was observed lower (*p* < 0.05) in Z and Y5Z silages compared to that in CK and Y5 silages. Chao 1 and Ace indices were decreased because of LAB or maize meal addition in relative to CK silage, and Y5Z silage presented the lowest (*p* < 0.05) Chao1 index. During ensiling process, microbes on the plants were mostly inhibited because of the anaerobic-acidic condition and were substituted by LAB (Sun J. J. et al., 2023). Therefore, a noticeable decrease in bacterial abundance was observed due to these transformations. Clear differences in diversity indices (Shannon and Simpson) were observed, as Y5 silage showed the highest (*p* < 0.05) bacterial diversity, followed by CK and Z silages, while Y5Z silage showed the lowest (*p* < 0.05) diversity. In agreement with our results, Li et al. (2020) reported that the bacterial diversity decreased in LAB or sucrose treated alfalfa silages due to the increased relative abundance of dominant *Lactobacillus*.

**Table 4:** Bacterial alpha diversity of *Orychophragmus violaceus* silages with or without LAB and maize meal.

Item	CK	Y5	Z	Y5Z	SEM	*p*-value
Coverage	0.997	0.997	0.998	0.998	0.0002	0.084
Richness estimator						
Sobs	140a	145a	51b	50b	15.2	0.002
Ace	354.25	263.44	207.54	227.10	24.741	0.146
Chao1	262.11a	200.38ab	171.42ab	118.12b	19.659	0.036
Diversity index						
Shannon	1.55b	2.13a	0.68c	0.38d	0.211	<0.001
Simpson	0.33c	0.18d	0.67b	0.84a	0.080	<0.001

Values with different letters in the same rows are significantly different at level of *p* < 0.05.

**Figure 1:**
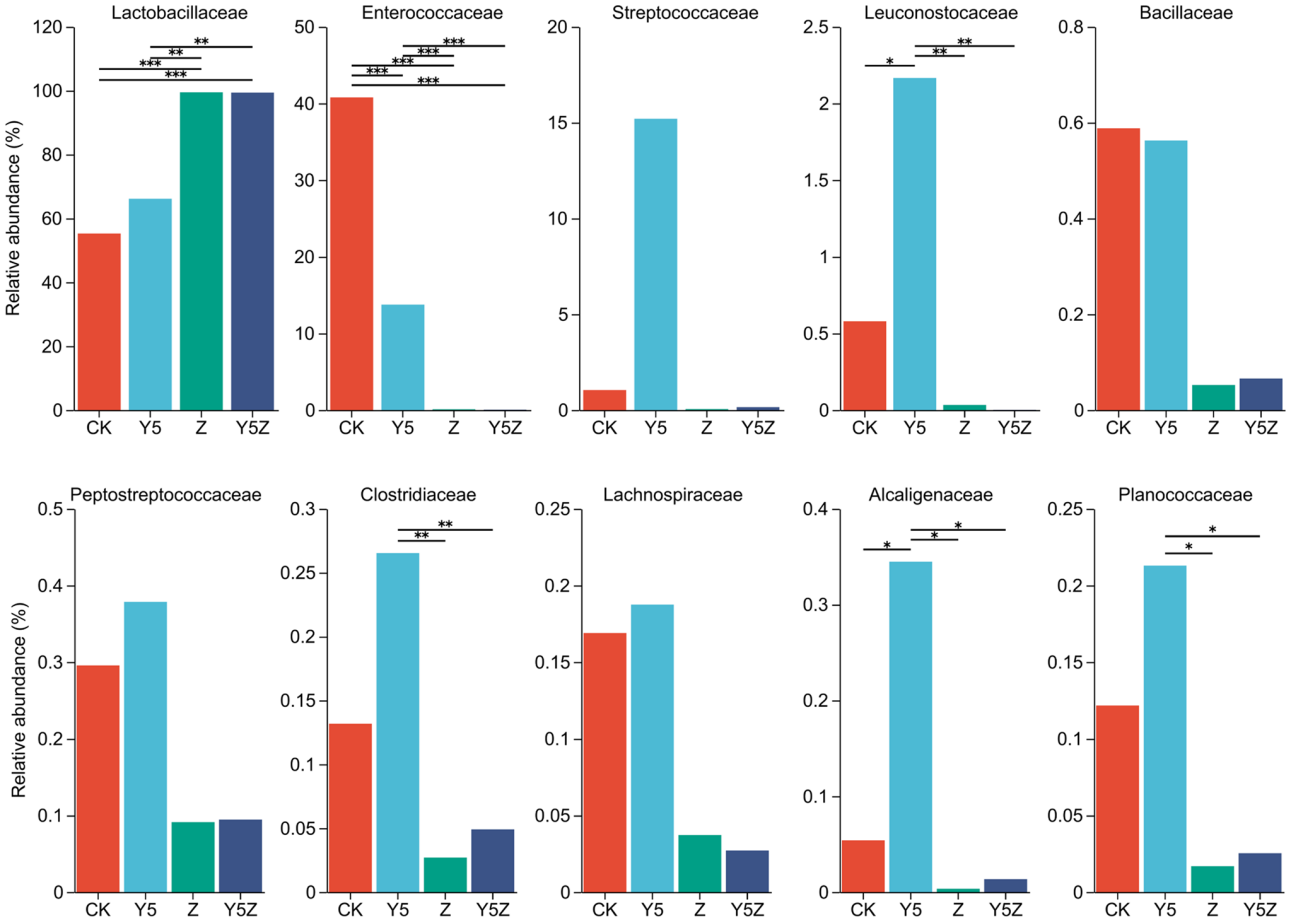
The family level bacterial taxa compositions of *Orychophragmus violaceus* silages with or without LAB and maize meal. CK, control; Y5, with maize meal; Z, with LAB; Y5Z, with LAB and maize meal. *, **, and *** stand for 0.01 < *p* < 0.05, 0.001 < *p* < 0.01, *p* < 0.001, respectively.

**Figure 2:**
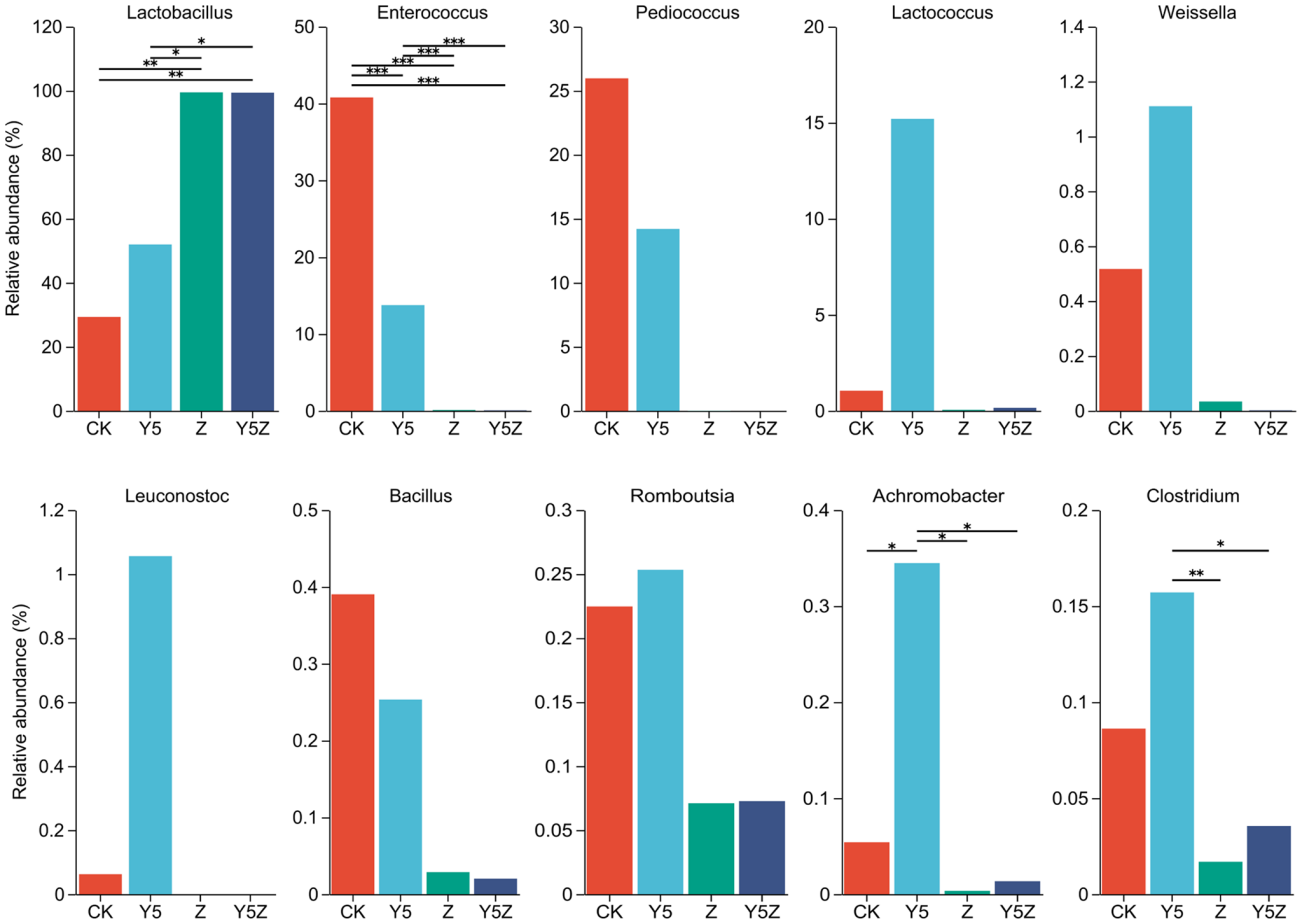
The genus level bacterial taxa compositions of *Orychophragmus violaceus* silages with or without LAB and maize meal. *, **, and *** stand for 0.01 < *p* < 0.05, 0.001 < *p* < 0.01, *p* < 0.001, respectively.

The top ten dominant bacteria at the family and genus levels in *O. violaceus* silage are shown in Figures 1, 2. Multiple comparisons indicated that the relative abundance varied across the different treatments. Similar to previous results found in silages prepared from alfalfa (Li et al., 2022; Sun J. J. et al., 2023), barley (Franco et al., 2022) and rice straw (Sun H. et al., 2023), the bacterial community observed in *O. violaceus* silage was dominated by Lactobacillaceae, mainly members of *Lactobacillus* and *Pediococcus*. In addition, the abundance of Lactobacillaceae and *Lactobacillus* was higher (*p* < 0.05) in Z and Y5Z silages than that in CK and Y5 silages.

According to the classical theory of ensiling fermentation, LA-producing cocci (*Weissella*, *Leuconostoc*, *Pediococcus*, *Lactococcus*, and *Enterococcus*) initiates LA fermentation at the early stage of ensiling, while LA-producing rod *Lactobacillus*, with acid tolerance ability, plays an important role in pH reduction at the later stage of ensiling (Jiang et al., 2020). The tendency of Enterococcaceae abundance was CK > Y5 > Z and Y5Z (*p* < 0.05), and the main genus was *Enterococcus*. In agreement with results observed in natural fermented soybean silage (Ni et al., 2017), *Enterococcus* was the dominant genus in CK silage followed by *Pediococcus* and *Lactobacillus*. During ensiling fermentation, *Enterococcus* played a pivotal role in accelerating the LA fermentation and building an anaerobic-acidic circumstance for the development of *Lactobacillus* (Wang et al., 2020; Dong et al., 2023). Recently, *Enterococcus* sp., such as *E. faecalis* and *E. faecium*, were successfully used as silage inoculants to stimulate ensiling process (Li et al., 2018). Similar tendency to *Enterococcus* was found for *Pediococcus*, although no significant difference was observed among treatments.

High relative abundance of LA-producing cocci, including *Enterococcus* (13.74%), *Pediococcus* (14.20%), *Lactococcus* (15.20%), *Weissella* (1.11%) and *Leuconostoc* (1.06%), was also observed in Y5 silage, although maize meal contributed to the growth of *Lactobacillus* (51.95%). The probable reason for the occurrence of abundant LA-producing cocci in CK and Y5 silages was that high moisture content partly compensate for acid sensitivity of LA-producing cocci as well as the limited competitiveness of native homofermentative *Lactobacillus* in *O. violaceus*. In addition, based on the concept proposed by Li et al. (2022), an unfinished or incomplete fermentation might occur in CK and Y5 silages as indicated by the high presence of LA-producing cocci. From a practical perspective, when reducing the moisture content of raw materials is not feasible or economical, homofermentative *Lactobacillus* strains that not only grow fast but also have other competitive advantages over their fellow LAB as well as other epiphytic bacteria were suggested to be applied in *O. violaceus* silage preparation to accelerate the ensiling process, and then to decrease the nutrient loss.

*Clostridium* is the main contributor for clostridial fermentation which not only causes reduction in nutritional value but also decreases hygienic quality of legume silage (Zheng et al., 2017). The relative abundance of *Clostridium* in Y5 silage was numerally higher than that in CK silage, and was higher (*p* < 0.05) than that in Z and Y5Z silages. The chances of a clostridial fermentation can be minimized by decreasing forages moisture content and inducing a rapid production of LA, because Clostridia are intolerant of both high osmotic pressure and low pH (Kung et al., 2018). In this study, however, maize meal might provide sufficient fermentable substrates for spoilage Clostridia growth in Y5 silage, when low pH alone did not necessarily depress growth of potentially undesired bacteria (Eikmeyer et al., 2013). It was suggested that potential positive effects of ensiling fermentation on inhibition deleterious microorganisms might be due to a direct effect of rapid acidification, reduced availability of water, production of antimicrobial compounds, or a combination of factors (Garde et al., 2014). *Achromobacter*, the main genus of family Alcaligenaceae, is non-fermentative and potential pathogen bacteria found ubiquitously in environmental reservoirs including rivers, ponds, residential water sources, soil, mud and some plants (Price et al., 2020). Similar to *Clostridium*, the relative abundance of undesirable *Achromobacter* was also observed higher (*p* < 0.05) in Y5 silage than other three groups.

**Figure 3:**
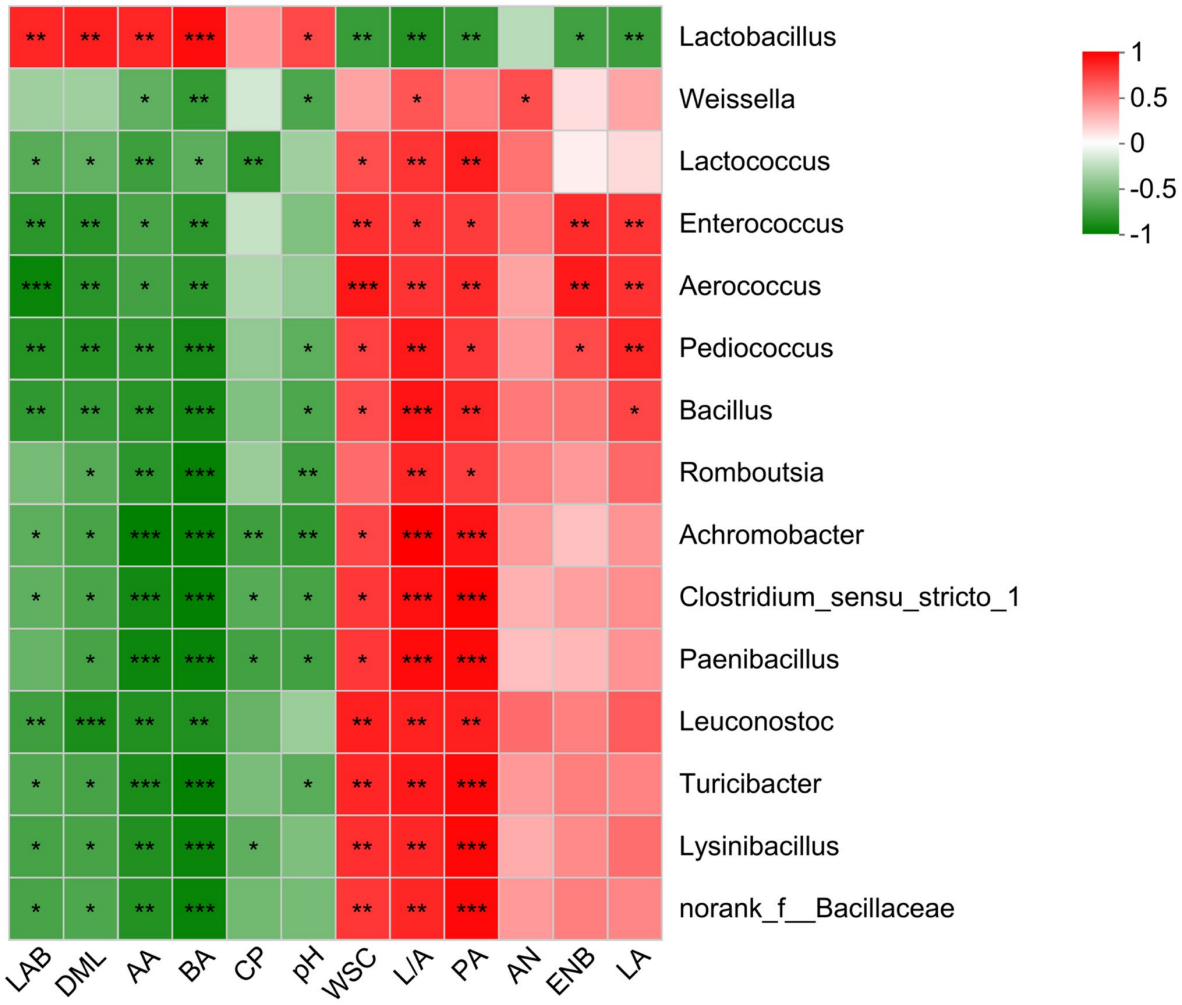
Spearman correlations between bacterial communities and fermentation parameters of *Orychophragmus violaceus* silages with or without LAB and maize meal. LAB, lactic acid bacteria; DML, dry matter loss; AA, acetic acid; BA, butyric acid; CP, crude protein; WSC, water-soluble carbohydrates; L/A, lactic to acetic acid ratio; PA, propionic acid; AN, ammonia nitrogen; ENB, Enterobacteria; LA, lactic acid. The corresponding value of the right heatmap is the correlation coefficient r, which ranges between −1 and 1. Red squares indicate a positive correlation, and green squares indicate a negative correlation. *, **, and *** stand for 0.01 < *p* < 0.05, 0.001 < *p* < 0.01, *p* < 0.001, respectively.

A Spearman correlation was performed to identify the relationship between fermentation parameters and top 15 genera in *O. violaceus* silage (Figure 3). During desirable ensiling process, epiphytic LAB converts WSC into LA resulting in decreased silage pH, thereby, results of correlation analysis usually reveal that *Lactobacillus* was positively correlated to LA and negatively correlated to WSC and pH. In this study, *Lactobacillus* was positively (*p* < 0.05) correlated to LAB, DML, AA, BA and pH, and negatively (*p* < 0.05) correlated to WSC, PA, Enterobacteria, LA and lactic to acetic acid ratio. Although the correlation was not always significant, LA-producing cocci was positively correlated to LA, and negatively correlated to pH. These results suggested that most of *Lactobacillus* in *O. violaceus* silage possessed a heterofermentative pathway and LA-producing cocci was the main contributor to LA production. Silage with clostridial fermentation is characterized by trace amount of LA and resultantly high levels of pH, BA, NH_3_−N and amines (Pahlow et al., 2003). In this study, *Clostridium* was negatively (*p* < 0.05) correlated to BA although positive correlation was observed for NH_3_−N production. This was partly consistent with the report of Franco et al. (2022), who found that BA was positively correlated to *Leuconostoc* and *Lactococcus*. It was not surprising because different species of the one genus, even if the one species existed in different habitats, contain different phylogenetic and basic characteristics (Huang et al., 2023). Hence, bacterial community in *O. violaceus* silage could potentially be further studied at species level and metabolic capability.

## 4. Conclusion

This study confirmed the potential of ensiling of *O. violaceus* as silage, the sufficient WSC enabled a large amount of LA production resulting in a rapid reduction in pH value. The growth of LA-producing cocci in CK silage was facilitated for good preservation of silage nutrients with low levels of DML and BA. However, exogenous LAB and maize meal generally provided little positive effect. Y5 silage had potential health risks for humans and animals as seen by frequent occurrence of pathogenic bacteria *Clostridium* and *Achromobacter*. AA, BA, and DML were increased dramatically in Z and Y5Z silages, indicating that the dominant *Lactobacillus* possessed a heterofermentative pathway and the *L. plantarum* strain composed in LAB inoculant was not suitable for *O. violaceus* silage. Homofermentative *Lactobacillus* strains, may be origin from *O. violaceus* plant, will be screened to further enhance ensiling process of *O. violaceus* silage.

## Data availability statement

The datasets presented in this study can be found in online repositories. The names of the repository/repositories and accession number(s) can be found in the article/supplementary material.

## Author contributions

MZ: Writing – review & editing, Funding acquisition, Investigation, Writing – original draft. PM: Writing – review & editing, Data curation. XT: Data curation, Writing – review & editing. LM: Writing – review & editing, Supervision.

## Funding

The author(s) declare financial support was received for the research, authorship, and/or publication of this article. This work was supported by the National Natural Science Foundation of China (32201461) and the Youth Research Fund of BAAFS (QNJJ202310).

## Conflict of interest

The authors declare that the research was conducted in the absence of any commercial or financial relationships that could be construed as a potential conflict of interest.

## Publisher’s note

All claims expressed in this article are solely those of the authors and do not necessarily represent those of their affiliated organizations, or those of the publisher, the editors and the reviewers. Any product that may be evaluated in this article, or claim that may be made by its manufacturer, is not guaranteed or endorsed by the publisher.

## Footnotes

^1^ https://www.majorbio.com/
